# Non–small cell lung cancer and immune checkpoint inhibitor therapy: does non-alcoholic fatty liver disease have an effect?

**DOI:** 10.1186/s12885-024-12295-6

**Published:** 2024-04-27

**Authors:** Yi Li, Bingxin Gong, Yusheng Guo, Weiwei Liu, Chao Chen, Shanshan Jiang, Feng Pan, Jiyu Song, Lian Yang, Guofeng Zhou

**Affiliations:** 1 Department of Radiology, Union Hospital, Tongji Medical College, Huazhong University of Science and Technology, No.1277 Jiefang Avenue, Wuhan, 430022 China; 2 Hubei Key Laboratory of Molecular Imaging, Wuhan, 430022 China; 3 Department of Pathology, Union Hospital, Tongji Medical College, Huazhong University of Science and Technology, Wuhan, 430022 China

**Keywords:** Non-small cell lung cancer, Immunotherapy, Immune checkpoint inhibitors, Non-alcoholic fatty liver disease, Immuno-inflammatory responses

## Abstract

**Background:**

Immunotherapy based on the application of immune checkpoint inhibitors (ICIs) is one of the standard treatments for advanced non-small cell lung cancer (NSCLC). Non-alcoholic fatty liver Disease (NAFLD) has demonstrated predictive value for response to immunotherapy in non-lung cancer types. Our study investigated the effect of NAFLD on the efficacy of real-life use of ICIs for patients with stage III / IV NSCLC.

**Methods:**

The clinical and imaging data of patients with stage III / IV NSCLC who were first admitted to Union Hospital, Tongji Medical College, Huazhong University of Science and Technology from March 2020 to July 2022 were retrospectively collected to ensure that they underwent at least one CT scan before treatment. A total of 479 patients were divided into the NAFLD group (Liver/Spleen density ratio ≤ 1) and the non-NAFLD group (Liver/Spleen density ratio > 1) by measuring the baseline liver and spleen CT value. The overall survival (OS), progression-free survival (PFS), objective response rate (ORR) and disease control rate (DCR) of the patients were obtained.

**Results:**

A total of 118 patients with NAFLD and 361 patients without NAFLD were included in the study. Patients with NAFLD tended to have higher BMI and higher total bilirubin compared to patients without NAFLD. The median duration of follow-up in the study was 22 m (IQR, 17–29 m). Both of 2 groups had a higher DCR (94% vs. 92%, *p* = 0.199) and ORR (38.1% vs. 44.9%, *p* = 0.452) respectively. There was no difference in efficacy between the two groups. In univariate analysis, NAFLD had no significant effect on PFS (*p* = 0.785) and OS (*p* = 0.851). Surprisingly, the presence of hypertension was observed to be associated with a higher OS (HR 1.471 95%CI 1.018–2.127, *p* = 0.040). Besides, based on multivariate analysis, lactic dehydrogenase was associated with PFS (HR 1.001 95%CI 1.000,1.002, *p* = 0.037) and OS (HR 1.002, 95%CI 1.001–1.003, *p* < 0.001).

**Conclusions:**

Among patients with NSCLC, NAFLD did not result in changes in survival or disease progression after immune checkpoint inhibitor therapy.

**Supplementary Information:**

The online version contains supplementary material available at 10.1186/s12885-024-12295-6.

## Introduction

Over the past decade, the introduction of immunotherapy has had a profound impact on the outlook for individuals diagnosed with non-small cell lung cancer (NSCLC) [1]. Immune checkpoint inhibitors (ICIs) such as PD-1 and PD-L1 have emerged as key therapeutic agents by obstructing the inhibitory pathways that normally regulate immune responses. By doing so, they reinstate an immune system that actively targets cancer cells, representing a critical advance in the treatment of advanced NSCLC [2]. Remarkably, these medications have demonstrated unprecedented enhancements across various clinical efficacy measures for patients with locally advanced or metastatic NSCLC [3]. Compared with traditional chemotherapy drugs, ICI improves patient survival outcomes while causing fewer adverse reactions, and has become an integral part of treatment algorithms [4].

Fatty liver disease, resulting from imbalanced metabolism due to unhealthy dietary habits and irregular lifestyles, has emerged as the most prevalent chronic liver disease worldwide [5]. Currently, non-alcoholic fatty liver disease (NAFLD) affects approximately 25% of the global adult population, with a particularly high incidence of 27% among Asian populations [6]. NAFLD is not just a liver disease, but it is also closely linked to systemic metabolic disorders and immune system abnormalities. Recent investigations have suggested that NAFLD is associated with alterations in the immune microenvironment, potentially influencing immune responses [7]. Patients with NAFLD often have comorbidities such as obesity, hypertension, and diabetes, which are known to be associated with immune dysfunction. There are also studies suggesting that the liver microenvironment may play a key role in NAFLD. The liver provides a unique pro-inflammatory microenvironment composed of a variety of immunoreactive cells, including Kupfer cells (KCs), T cells, antigen-presenting cells (APC), and hepatic stellate cells (HSC) [8]. These cells possess the capacity to augment the immune response and expedite the progression of liver fibrosis and damage. In addition to this, these immune cells secrete various bioactive factors, such as pro-inflammatory cytokines, leptin, bile acids, and free fatty acids, which effectively interact with the constituents of the liver microenvironment, precipitating inflammation, fibrosis, and lipid toxicity. The coexistence of metabolic dysfunction caused by NAFLD and alterations in the gut microbiota may collaborate to stimulate innate and adaptive immune responses, ultimately promoting both hepatic and systemic inflammatory reactions [9]. Notably, a recent study demonstrated that NAFLD could attenuate the efficacy of immunotherapy in hepatocellular carcinoma [10]. In this study, a preclinical model of hepatocellular carcinoma induced by non-alcoholic steatohepatitis was employed as the experimental model. Utilizing therapeutic immunotherapy aimed at PD1, a notable augmentation of activated CD8( +) PD1( +) T cells within the tumor milieu was observed. However, despite this immune response, tumor regression did not occur, implying a compromised functionality of tumor immune surveillance. Previous research has explored the relationship between NAFLD and the immune system, prompting consideration of whether NAFLD impacts immune checkpoint inhibitor treatment. To date, no studies have assessed the impact of NAFLD on the real-world efficacy outcomes of first-line ICI therapy for NSCLC in routine clinical practice. Therefore, our retrospective study aims to investigate the disparity in real-world treatment efficacy of ICIs in patients with and without NAFLD.

## Methods and materials

Prior to the start of the study, our study was approved by the Ethics Committee of Union Hospital, Tongji Medical College, Huazhong University of Science and Technology (Institutional Review Board No. S054).

### Study participants and design

This was a single-center retrospective cohort study. From March 2020 to July 2022, we included 479 with NSCLC who received immunotherapy at the Union Hospital, Tongji Medical College, Huazhong University of Science and Technology. All participants enrolled in this study were (a) histologically or cytologically confirmed NSCLC; (b) diagnosed with stage III or IV NSCLC following NCCN Clinical Practice Guidelines in Oncology: NSCLC (Version 3.2022); [11] (c) older than 18 years of age; (d) received at least three cycles of immunotherapy; (e) diagnosed and treated at our institution. Participants for both groups were excluded if they (a) had other types of tumors; (b) received surgical treatment; (c) had other liver diseases (e.g., alcoholic fatty liver, alcoholic hepatitis, liver fibrosis, cirrhosis and drug-induced liver injury); (d) with history of excessive drinking (> 210 g/week for men and > 140 g/week for women in the past 12 months); [12] (e) whose medical record is incomplete. (f) were not initially treated with immunotherapy. Before the onset of the study, approval from the Ethics Committee of the Tongji Medical College of Huazhong University of Science and Technology. Before the onset of the study, approval from the Institutional Ethics Committee and informed consent from each subject were obtained.

### Data acquisition and analysis

All CT images were obtained using a Philips Healthcare scanner. The acquisition settings included a tube voltage of 120kVp, with the tube current automatically regulated. The images were reconstructed with a matrix size of 512 × 512 and a collimation of 64 × 0.625 mm. The reconstructed slice thickness and interval were both set at 1.5 mm. To assess the CT values of the tissues, a measurement tool integrated within the Picture Archiving and Communication System (PACS) was utilized.

Two experienced radiologists (YL and BXG) performed measurements in the right lobe of the liver and at the location of the spleen at the same level, and then delineated round regions of interest (About 120mm^2^), avoiding bile ducts, blood vessels and artifacts (Supplementary Fig. 1). The measurements were made by the two aforementioned radiologists, and the average of the measurements was used as the final result. According to whether the calculated Liver/Spleen density ratio (L/S ratio) was less than 1, the patients were divided into patients with NAFLD and non-NAFLD. Density ratio is considered to be a reliable imaging index for the diagnosis of NAFLD [13]. Fifty patient images were randomly selected and segmented on the designated slice by a radiologist (YL) after a two-week interval, and another radiologist (GFZ) with 31 years of experience independently performed the same task to assess the repeatability of feature extraction both within and between observers. The intraclass correlation coefficient (ICC) was subsequently employed to evaluate the agreement in imaging evaluation within and between observers. The obtained ICC value of 0.94 indicates minimal differences among individuals within the group. The observers were blinded to all clinical data, and any discrepancies were resolved through consensus.

### Patient outcomes

Patients’ baseline demographics and clinical outcomes were collected retrospectively. The radiological response following treatment with ICIs was assessed as per RECIST criteria v1.1 [14] on CT performed. Overall survival (OS) was defined as the time from the first dose to death or last follow-up. Progression-free survival (PFS) was defined as the time from the first dose to radiographic imaging progression or death, whichever occurred first. The Overall response rate (ORR) included patients with complete response (CR) and partial response (PR). The Disease control rate (DCR) included all patients who achieved CR, PR, or stable Disease (SD). Progressive disease (PD) included all patients with radiographic evidence of intrapulmonary progression or extrapulmonary spread.

### Statistical analysis

For relevant analysis, patients were divided into two cohorts. The principle we followed was as follows: L/S ratio was defined as the CT value of the spleen divided by the CT value of the liver. Patients were divided into those with L/S ratio of less than or equal to 1 (NAFLD) and those with L/S ratio of more than 1 (Non-NAFLD). Baseline characteristics were compared within the divided L/S ratio (NAFLD vs. Non-NAFLD). The χ2 test was used to compare categorical data and the unpaired student t-test for continuous data. DCR and ORR were compared using Fisher’s exact test. To eliminate redundant and unstable features, ICC calculation and Pearson’s correlation analysis were conducted.

We employed the Kaplan–Meier method to conduct time-to-event analysis for OS and PFS. The OS and PFS were compared between patient cohorts with and without non-alcoholic fatty liver disease (NAFLD) using the log-rank test. A *p*-value of less than 0.05 was considered statistically significant. To further evaluate the impact of NAFLD on patient survival, univariate and multivariate Cox regression models were constructed. Parameters with a *p*-value less than 0.10 in the univariate analysis were included in the multivariate Cox regression model to calculate the hazard ratio (HR) and its corresponding 95% confidence interval (95% CI). Furthermore, we conducted exploratory subgroup analyses based on clinically relevant covariates and reported HRs with their respective 95% CIs within each subgroup.

## Results

### Baseline characteristics

Among the 479 patients with advanced NSCLC (361 NAFLD, 118 Non-NAFLD) included in this analysis, the median age was 64 years (IQR, 58–69 years), and the median BMI was 22.23 kg/m^2^ (IQR, 20.08–24.22 years). The baseline characteristics of patients are shown in Table 1. No significant differences in gender (*p* = 0.064), age (*p* = 0.780), diabetes (*p* = 0.289), hypertension (*p* = 0.560) and so on.

**Table 1:** Baseline characteristics of the study population stratified according to the presence or absence of nonalcoholic fatty liver disease

Characteristics	Non-NAFLD	NAFLD	*P* value
Patient characteristics			
Patients, *n*	361	118	
Gender, *n* (%)			0.064
Male	310 (64.7%)	109(22.8%)	
Female	51 (10.6%)	9 (1.9%)	
Age, No. (%)			0.780
< 65	195 (40.7%)	62 (12.9%)	
≥ 65	166 (34.7%)	56 (11.7%)	
Body mass index (kg/m^2^)*, *n* (%)			< 0.001
≤ Median	308 (64.3%)	83 (17.3%)	
> Median	53 (11.1%)	35 (7.3%)	
ECOG performance status			0.983
0	135 (28.2%)	44 (9.2%)	
≥ 1	226 (47.2%)	74 (15.4%)	
Diabetes, *n* (%)	31 (6.5%)	14 (2.9%)	0.289
Hypertension, *n* (%)	110 (23%)	42 (8.8%)	0.299
Smoking, *n* (%)	190 (39.7%)	70 (14.6%)	0.205
Hyperlipidemia, *n* (%)	109 (22.8%)	39 (8.1%)	0.560
Hemoglobin (g/L), mean (SD)	123.7 (16.2)	124.4 (16.4)	0.674
Blood urea nitrogen (mmol/L), mean (SD)	5.3 (1.7)	5.6 (1.9)	0.105
Serum creatinine (umol/L), mean (SD)	72.0 (14.7)	73.0 (15.0)	0.530
Total bilirubin (mg/dL), mean (SD)	10.5 (4.8)	12.8 (6.1)	< 0.001
Alanine aminotransferase (U/L), mean (SD)	24.5 (28.3)	27.78 (26.9)	0.281
Aspartate aminotransferase (U/L), mean (SD)	23.4 (14.9)	26.4 (28.9)	0.139
Alkaline phosphatase (U/L), mean (SD)	100.7 (59.1)	102.8 (49.4)	0.733
γ-glutamyl transpeptadase (U/L), mean (SD)	47.4 (68.4)	58.0 (60.4)	0.133
Albumin (g/L), mean (SD)	38.6 (4.9)	37.6 (4.5)	0.058
Albumin/Globulin ratio, mean (SD)	1.5 (1.2)	1.3 (0.3)	0.144
lactic dehydrogenase (U/L), mean (SD)	236.9 (115.8)	229.2 (95.8)	0.516
Pathological types, *n* (%)			0.540
adenocarcinoma	169 (35.3%)	50 (10.4%)	
squamous carcinoma	169 (35.3%)	62 (12.9%)	
other	23 (4.8%)	6 (1.3%)	
Types of ICIs, *n* (%)			0.727
PD-1 antibody	333 (69.5%)	110 (23%)	
PD-L1 antibody	28 (5.8%)	8 (1.7%)	
Stages, *n* (%)			0.998
Stage III	260 (54.3%)	85 (17.7%)	
Stage IV	101 (21.1%)	33 (6.9%)	

*Abbreviation: NAFLD* Patients with non-alcoholic fatty liver disease, *Non-NAFLD* Patients without non-alcoholic fatty liver disease, *SD* Standard deviation, *ICIs* Immune checkpoint inhibitors, *PD-1* programmed cell death-1, *PD-L1* Programmed cell death-ligand 1

^*^Median body mass index is 25 kg/m^2^

Rates of NAFLD were similar between the gender groups (*p* = 0.064). Two hundred and twenty-two participants (46.3%) were 65 years or older at the time of enrollment, and two hundred and fifty-seven participants (53.7%) younger. Turns out, NAFLD occurred in comparable proportions between younger and older individuals (*p* = 0.780). Patients with NAFLD had a higher median BMI (median 22.895 vs. 22.090 kg/m^2^, *p* < 0.001) than patients without NAFLD, which is common sense. Besides, participants with NAFLD also had higher total bilirubin (mean 12.8 vs. 10.5,* p* < 0.001).

### Efficacy

**Fig. 1:**
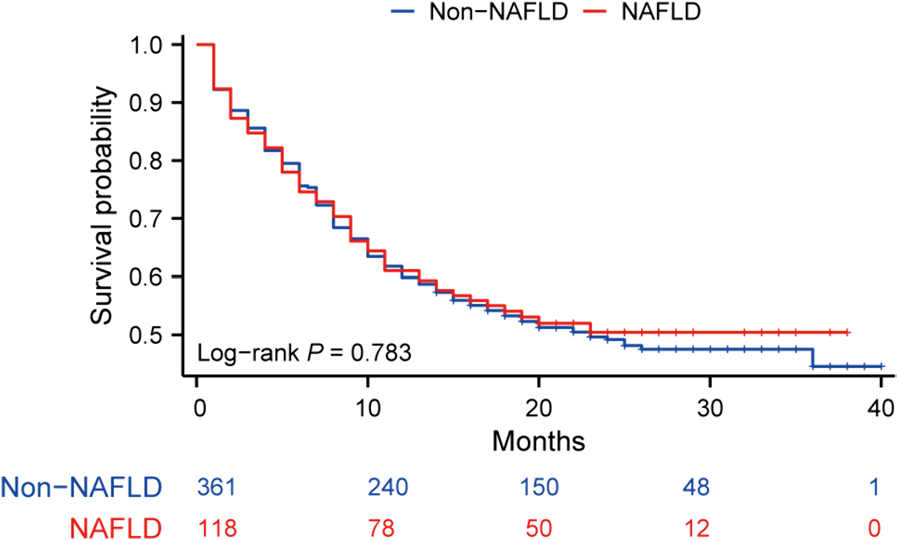
Kaplan–Meier curve showing progression-free survival for patients with and without non-alcoholic fatty liver disease

**Fig. 2:**
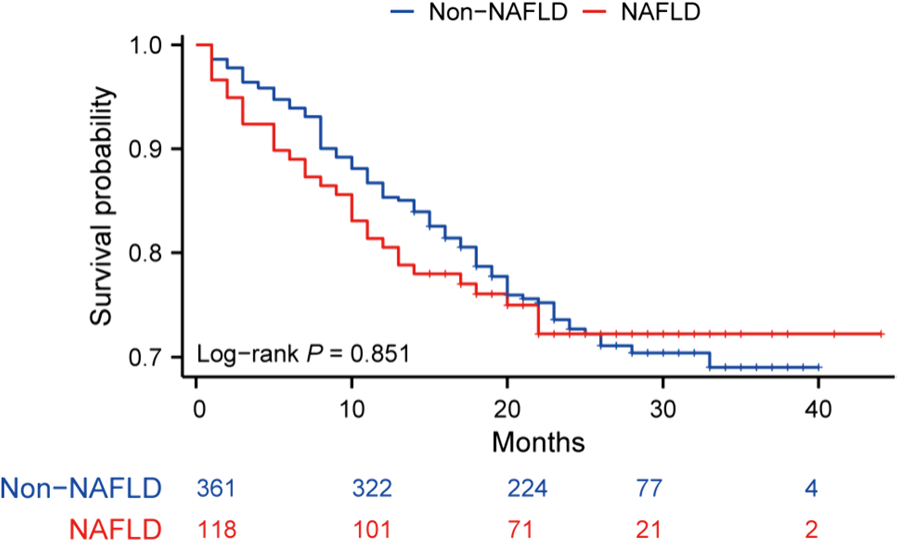
Kaplan–Meier curve showing overall survival for patients with and without non-alcoholic fatty liver disease

At the time of analysis, 128 (26.7%) patients had died. Because of the short follow-up, the median survival time was not reached. The median follow-up time was 22 m (IQR, 17–29 m). Survival curves were generated based on the available data. There was no statistically significant difference in PFS between NAFLD and non-NAFLD groups (*p* = 0.783), as was OS (*p* = 0.851) (Fig. 1, Fig. 2).

All patients were assessed for tumor response according to RECIST v1.1 criteria. Patients with NAFLD had similar ORR (38.1% vs. 44.9%,* p* = 0.199) and DCR (94.0% vs.92.0%, *p* = 0.452) compared to patients without NAFLD (Supplementary Table 1). NAFLD did not have a significant effect on PFS in univariate analysis (HR 0.959, 95% CI 0.713–1.292, *p* = 0.785) (Table 2). OS was also consistent between the NAFLD cohorts (HR 1.039, 95% CI 0.694–1.558, *p* = 0.851) (Table 3). Age is an important factor affecting the efficacy of treatment, and the efficacy of older patients is lower than that of younger patients. In addition, our results validated that the low short-term response and survival rates in lung cancer patients are related to the stage of lung cancer at initial diagnosis [15]. Surprisingly, the presence of hypertension was observed to be associated with a higher OS (HR 1.471 95%CI 1.018–2.127, *p* = 0.040). Besides, based on multivariate analysis, lactic dehydrogenase was associated with PFS (HR 1.001 95% CI 1.000–1.002,* p* = 0.037) and OS (HR 1.002 95%CI 1.001–1.003, *p* < 0.001) (Table 2, Table 3).

**Table 2:** Effects of non-alcoholic fatty liver disease and prognostic factors on progression-free survival after Immune checkpoint inhibitor therapy in univariate and multivariate Cox regression models

Parameter	Univariate analysis		Multivariate analysis	
	Hazard ratio (95% CI)	*P* value	Hazard ratio (95% CI)	*P* value
Gender				
Male	Reference			
Female	1.072 (0.735—1.563)	0.719		
Age				
< 65	Reference			
≥ 65	1.116 (0.866—1.437)	0.397		
Body mass index (kg/m^2^)*				
< Median	Reference			
≥ Median	0.867 (0.617—1.218)	0.410		
ECOG performance status				
0	Reference		Reference	
≥ 1	1.477 (1.144—1.906)	0.003	1.336 (1.033—1.728)	0.027
Diabetes				
No	Reference			
Yes	1.395 (0.934—2.082)	0.104		
Hypertension				
No	Reference			
Yes	0.947 (0.718—1.248)	0.698		
Smoking				
No	Reference			
Yes	0.844 (0.655—1.087)	0.189		
Hyperlipidemia				
No	Reference			
Yes	0.947 (0.718—1.248)	0.698		
Total bilirubin	1.001 (0.976—1.027)	0.924		
Albumin/Globulin ratio	0.582 (0.385—0.881)	0.011	0.628 (0.397—0.994)	0.047
lactic dehydrogenase	1.002 (1.001—1.003)	0.001	1.001 (1.000—1.002)	0.037
Hemoglobin (g/L)	0.989 (0.981—0.996)	0.004	0.994 (0.986—1.002)	0.138
Blood urea nitrogen	0.935 (0.867—1.008)	0.079	0.924 (0.858—0.995)	0.035
Serum creatinine	0.996 (0.987—1.004)	0.325		
Pathological types				
adenocarcinoma	Reference			
squamous carcinoma	0.782 (0.602—1.015)	0.064		
other	0.673 (0.380—1.193)	0.176		
Types of ICIs				
PD-1 antibody	Reference			
PD-L1 antibody	1.195 (0.756—1.889)	0.445		
Stages				
Stage III	Reference		Reference	
Stage IV	2.062 (1.490—2.852)	< 0.001	2.037 (1.461—2.840)	< 0.001
Groups				
Non-NAFLD	Reference			
NAFLD	0.959 (0.713—1.292)	0.785		

*Abbreviations: 95% CI* 95% Confidence Interval, *Non-NAFLD* Patients without non-alcoholic fatty liver disease, *NAFLD* Patients with non-alcoholic fatty liver disease, *ICIs* Immune checkpoint inhibitors, *PD-1* Programmed cell death-1, *PD-L1* Programmed cell death-ligand 1

^*^Median body mass index is 25 kg/m^2^

**Table 3:** Effects of non-alcoholic fatty liver disease and prognostic factors on overall survival after Immune checkpoint inhibitor therapy in univariate and multivariate Cox regression models

Parameter	Univariate analysis		Multivariate analysis	
	Hazard ratio (95% CI)	*P* value	Hazard ratio (95% CI)	*P* value
Gender				
Male	Reference			
Female	0.846 (0.485—1.473)	0.554		
Age				
< 65	Reference		Reference	
≥ 65	1.710 (1.204—2.429)	0.003	1.537 (1.061—2.227)	0.023
Body mass index (kg/m^2^)*				
< Median	Reference			
≥ Median	1.046 (0.671—1.631)	0.842		
ECOG performance status				
0	Reference		Reference	
≥ 1	1.874 (1.325—2.650)	< 0.001	1.654 (1.161—2.355)	0.005
Diabetes				
No	Reference			
Yes	1.522 (0.901—2.570)	0.116		
Hypertension				
No	Reference		Reference	
Yes	1.695 (1.193—2.408)	0.003	1.471 (1.018—2.127)	0.040
Smoking				
No	Reference			
Yes	0.972 (0.686—1.376)	0.871		
Hyperlipidemia				
No	Reference			
Yes	1.053 (0.726—1.527)	0.785		
Total bilirubin	1.022 (0.990—1.056)	0.179		
Albumin/Globulin ratio	0.485 (0.271—0.867)	0.015	0.636 (0.334—1.210)	0.168
lactic dehydrogenase	1.003 (1.002—1.004)	< 0.001	1.002 (1.001—1.003)	< 0.001
Hemoglobin (g/L)	0.987 (0.976—0.997)	0.014	0.993 (0.981—1.004)	0.199
Blood urea nitrogen	0.968 (0.875—1.071)	0.531		
Serum creatinine	1.005 (0.994—1.017)	0.382		
Pathological types				
adenocarcinoma	Reference			
squamous carcinoma	0.847 (0.594—1.207)			
other	0.542 (0.218—1.345)			
Types of ICIs				
PD-1 antibody	Reference			
PD-L1 antibody	0.917 (0.466—1.807)	0.803		
Stages				
Stage III	Reference		Reference	
Stage IV	1.921 (1.223—3.017)	0.005	1.800 (1.132—2.862)	0.013
Groups				
Non-NAFLD	Reference			
NAFLD	1.039 (0.694—1.558)	0.851		

*Abbreviations: 95% CI* 95% Confidence Interval, *Non-NAFLD* Patients without non-alcoholic fatty liver disease, *NAFLD* Patients with non-alcoholic fatty liver disease, *ICIs* Immune checkpoint inhibitors, *PD-1* Programmed cell death-1, *PD-L1* Programmed cell death-ligand 1

^*^Median body mass index is 25 kg/m^2^

In addition, the preliminary subcomponent analysis was carried out in order to explore the factors that could affect the curative effect (Supplementary Fig. 2, Supplementary Fig. 3). Unsurprisingly, we found no potentially positive results in the subgroup for PFS and OS.

## Discussion

This study has profound implications in the field of immunotherapy for lung cancer. The use of ICIs has become one of the most promising methods in the field of cancer treatment and has achieved satisfactory results in the clinical application of a variety of cancers [16]. Currently, due to its clear survival benefit, immunotherapy has become a standard first-line treatment modality for advanced NSCLC [17]. The population of patients with NAFLD is large, and the incidence is rising. Similarly, the proportion of lung cancer patients with underlying non-alcoholic fatty liver disease will grow. In our study, we chose the liver-spleen ratio as the assessment criterion for NAFLD because, firstly, the liver/spleen density ratio, measured with the use of CT scan, is a reliable, accurate, and noninvasive diagnostic tool for NAFLD [18]. It is considered to provide a useful noninvasive method for the detection and follow-up of patients with fatty liver when liver biopsy is contraindicated, [19] and it has been widely used in many studies [20, 21] and clinics. Second, CT-based liver/spleen density ratio is easier to measure than liver biopsy and MRI techniques. Especially in patients with lung cancer, almost every patient with lung cancer will undergo routine CT scan before treatment, which will scan the level of liver and spleen, and the liver/spleen density ratio can be easily obtained, which is practical and operable for clinicians. In the occurrence and progression of NAFLD, the immune system plays an integral role. [22] NAFLD has been shown to reduce tumor infiltration by immune cells in order to impair the ability of immunosuppressants to suppress tumor growth [23]. A recent study from the German Cancer Research Center showed that large numbers of abnormal CD8/PD-1 double-positive T cells accumulate in the liver in response to nonalcoholic steatohepatitis. If PD-1/L1 inhibitors activate these T cells, they will not only fail to kill the tumor but also aggravate liver tissue damage, making the therapeutic effect counterproductive [10]. They speculate that the same mechanism may occur in patients with other tumor types. A study by Pinto et al. showed that NAFLD may act as a potential factor affecting the immuno-tumor microenvironment in hepatocellular carcinoma, but different causes cause different responses. [24] However, a meta-analysis concluded otherwise [25]. On the contrary, we demonstrated NAFLD delivers no clinical benefit for patients with NSCLC who undergo ICI-based therapy, which is consistent with Zhou et al.’s research [26]. In addition, we validated that NAFLD did not have an impact on the survival time of patients in a larger cohort analysis. We speculated that NAFLD may induce changes in the components of the local immune microenvironment of the liver, aggravate liver injury and the progression of liver cancer, [27] but has a weak effect on the systemic immune system, which can only lead to a decline in the efficacy of immunotherapy in liver cancer, but the same performance cannot be seen in lung cancer. Nevertheless, complex regulatory mechanisms for the interaction of NAFLD with the immune system have not been fully defined, which is expected to be investigated in future studies.

At baseline, we found that NAFLD patients tended to have high total bilirubin and BMI. Generally, the proinflammatory mechanisms of NAFLD trigger high levels of total bilirubin [28]. The relationship of NAFLD and BMI mapped its relationship with obesity, which is complicated. Excess adiposity is often considered to be a manifestation of metabolic disorders associated with chronic systemic inflammation. Especially in the tumor microenvironment, it can cause an imbalance of the immune system [29]. Previous studies have shown that higher BMI seems to be associated with improved OS in patients with NSCLC after immunotherapy [30]. However, in univariate analysis, BMI was not verified to influence clinical benefit in our study. A recent mate analysis [31] shows that there is no evidence to prove a straight causal relationship between obesity and outcomes of ICIs, but sarcopenic obesity is a predictor of poor survival outcomes and toxicity in cancer patients receiving chemotherapy [32]. This may be because the parameters of body composition analysis are complex and need to be taken into consideration by multiple factors.

After performing multivariate analysis, we discovered that older patients predicted shorter survival, while patients with stage IV cancer tended to have a worse prognosis than those with stage III cancer, which is a well-known conclusion. The ECOG performance score (ECOG PS) is a widely used method for physicians to comprehensively assess patients’ symptoms and mobility. The higher the ECOG PS, the worse the patient’s physical condition. We observed that ECOG PS can predict the prognosis of patients with non-small-cell lung cancer treated with immune checkpoint inhibitors, consistent with Miao et al. [33] Patients with hypertension may have higher OS in patients after immunotherapy, which may be related to the immune-inflammatory mechanism of hypertension onset. Previous studies have shown that hypertension induces the increase of extracellular ATP, which induces the enhancement of APC and T cell function [34]. An alternative perspective focuses on prior treatment of hypertension. The drug α2 adrenoceptor agonists used in the treatment of hypertension can mediate the function of tumor-associated macrophages and CD4( +) T cells so that hypertensive patients with a history of related treatment can better benefit from immunotherapy [35]. Interestingly, lactate dehydrogenase appears to be associated with both long- and short-term survival in lung cancer patients. Our data suggested that baseline lactate dehydrogenase levels may be associated with the response to immunotherapy. Mezquita et al. [36] propose in a multicenter study that baseline lactate dehydrogenase levels are a marker of response to immunotherapy in patients with NSCLC. Many later studies have also reached similar conclusions, indicating that LDH may be an easily available indicator for predicting immune efficacy and has value in the stratified treatment of patients.

Our study has some limitations. First and foremost, this is a retrospective study subject to collection and selection bias. Although we can easily evaluate the L/S ratio on a plain CT scan of the lung, CT as a noninvasive assessment of NAFLD has relatively poor performance in detecting mild steatosis and quantitative steatosis [37]. In addition, the L/S ratio is not a gold standard for NAFLD diagnosis, liver biopsy data are needed to confirm whether NAFLD affects the efficacy of NSCLC immunotherapy ultimately. Additionally, although it represents the safety of immunotherapy, we did not record immune-related adverse events. Despite these limitations, to the best of our knowledge, this is the largest study assessing the efficacy of Immune checkpoint inhibitor therapy in patients with and without NAFLD. Therefore, prospective multi-center clinical trials are required to verify our results. In conclusion, NAFLD holds no clinical benefit for advanced NSCLC patients who undergo ICI-based treatment, but it is associated with improved outcomes in patients with LMs according to Zhou et al.’s study [26]. At the same time, we also look forward to relevant research in the future to support it.

## Conclusion

In summary, our study shows NAFLD does not affect immunotherapy efficacy in NSCLC patients treated with ICI.

### Supplementary Information

**Additional file 1:** **Supplementary Table 1 **Radiological response evaluated per RECIST criteria version 1.1 stratified according to the presence or absence of nonalcoholic fatty liver disease. **Supplementary Figure 1** Sample figure of the measurement of the liver/spleen ratio. A) A woman, 53 years old, did not suffer from non-alcoholic fatty liver disease. B) A man, 50 years old, suffered from non-alcoholic fatty liver disease. **Supplementary Figure 2 **Forest plot of progression-free survival.** Supplementary Figure 3 **Forest plot of overall survival.

## Data Availability

The raw data supporting the conclusions of this article will be made available by the authors, without undue reservation. If someone wants to request the date, he can contact the corresponding author by email, upon reasonable request.
