# Supplementary material for: Non–small cell lung cancer and immune checkpoint inhibitor therapy: does non-alcoholic fatty liver disease have an effect?
Source: BMC Cancer. 2024 Apr 27;24:535. doi: 10.1186/s12885-024-12295-6 (PMC11055283; doi:10.1186/s12885-024-12295-6)
Supplement: Supplementary file 1 — Additional file 1: Supplementary Table 1 Radiological response evaluated per RECIST criteria version 1.1 stratified according to the presence or absence of nonalcoholic fatty liver disease. Supplementary Figure 1 Sample figure of the measurement of the liver/spleen ratio. A) A woman, 53 years old, did not suffer from non-alcoholic fatty liver disease. B) A man, 50 years old, suffered from non-alcoholic fatty liver disease. Supplementary Figure 2 Forest plot of progression-free survival. Supplementary Figure 3 Forest plot of overall survival. [file 12885_2024_12295_MOESM1_ESM.docx]

Supplementary Material

**Supplementary Table 1** Radiological response evaluated per RECIST criteria version 1.1 stratified according to the presence or absence of nonalcoholic fatty liver disease.

|  | Non-NAFLD  (n=361) | NAFLD  (n=118) | *P* value |
| --- | --- | --- | --- |
| Complete response | 0 | 0 |  |
| Partial response | 162 (44.9%) | 45 (38.1%) |  |
| Stable disease | 170 (47.1%) | 66 (55.9%) |  |
| Progressive disease rate | 29 (8.0%) | 7 (6.0%) |  |
| Objective response rate | 44.9% | 38.1% | 0.199 |
| Disease control rate | 92.0% | 94.0% | 0.452 |

Abbreviation: RECIST, Response evaluation criteria in solid tumors; NAFLD, Patients with non-alcoholic fatty liver disease; Non-NAFLD, Patients without non-alcoholic fatty liver disease.

**
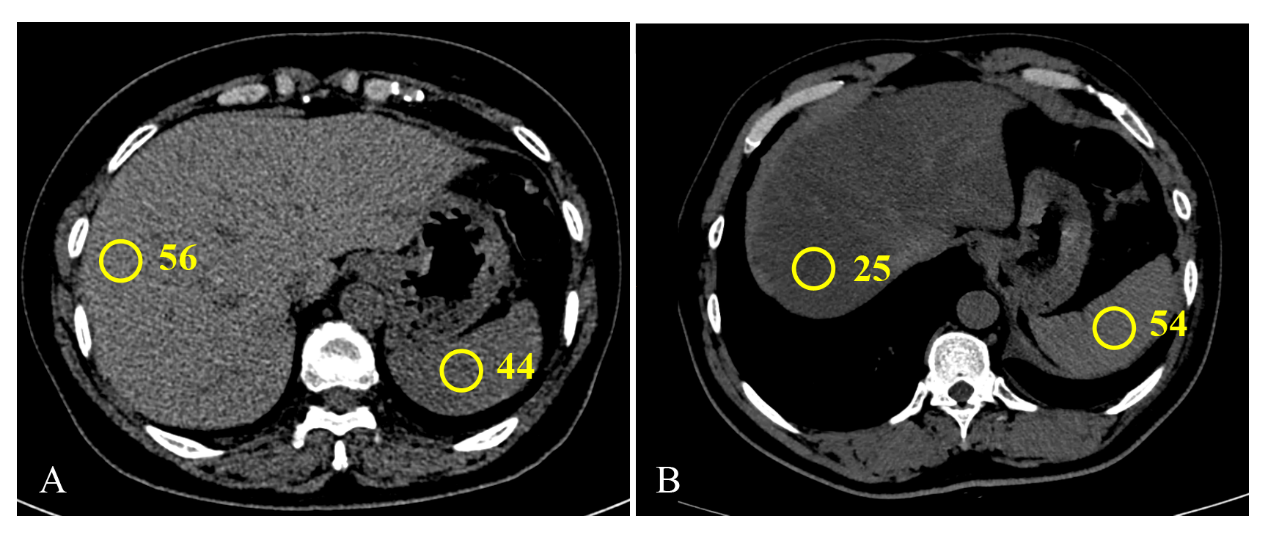
**

**Supplementary Figure 1** Sample figure of the measurement of the liver/spleen ratio. A) A woman, 53 years old, did not suffer from non-alcoholic fatty liver disease. B) A man, 50 years old, suffered from non-alcoholic fatty liver disease.


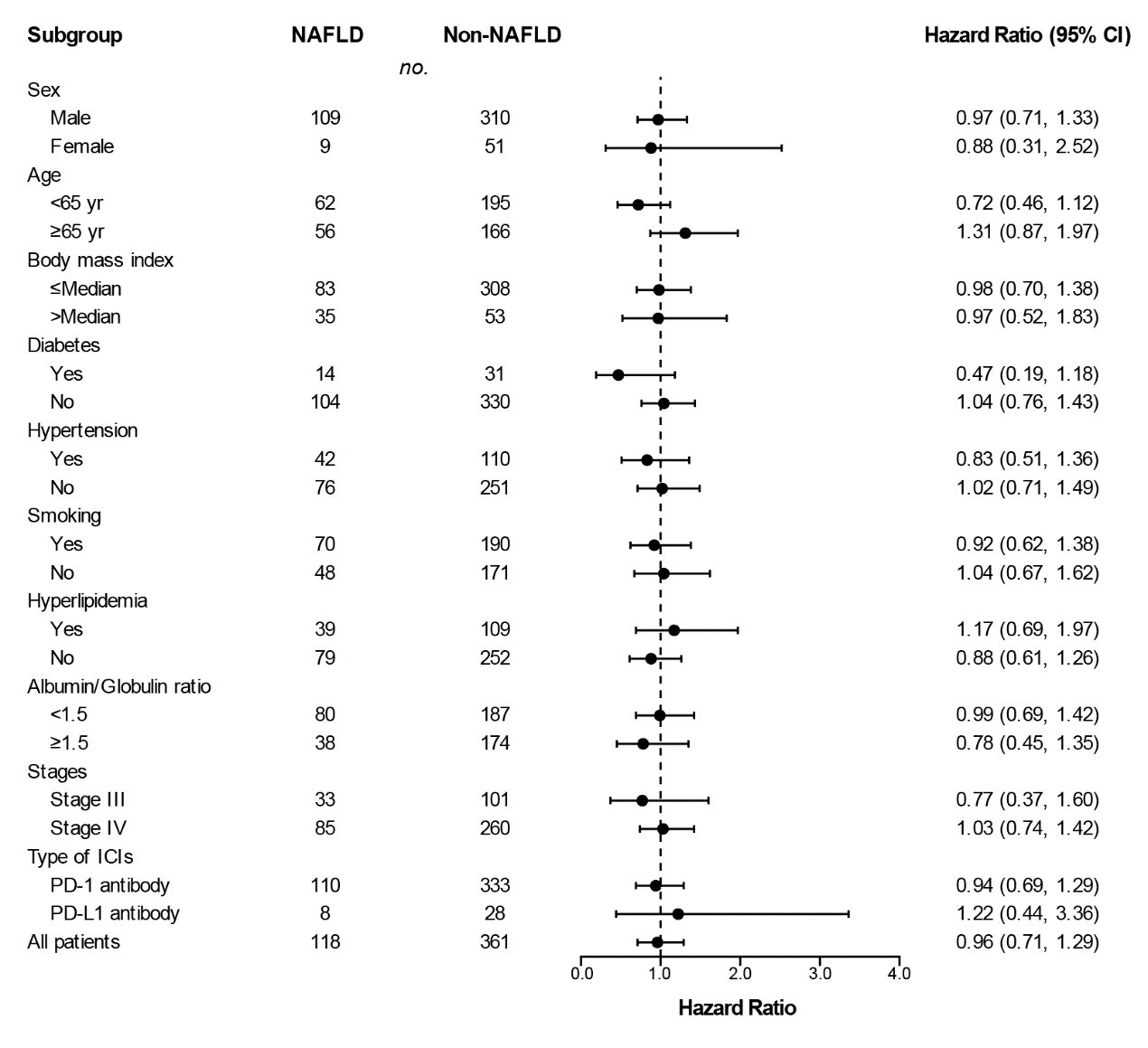


**Supplementary Figure 2** Forest plot of progression-free survival**.**


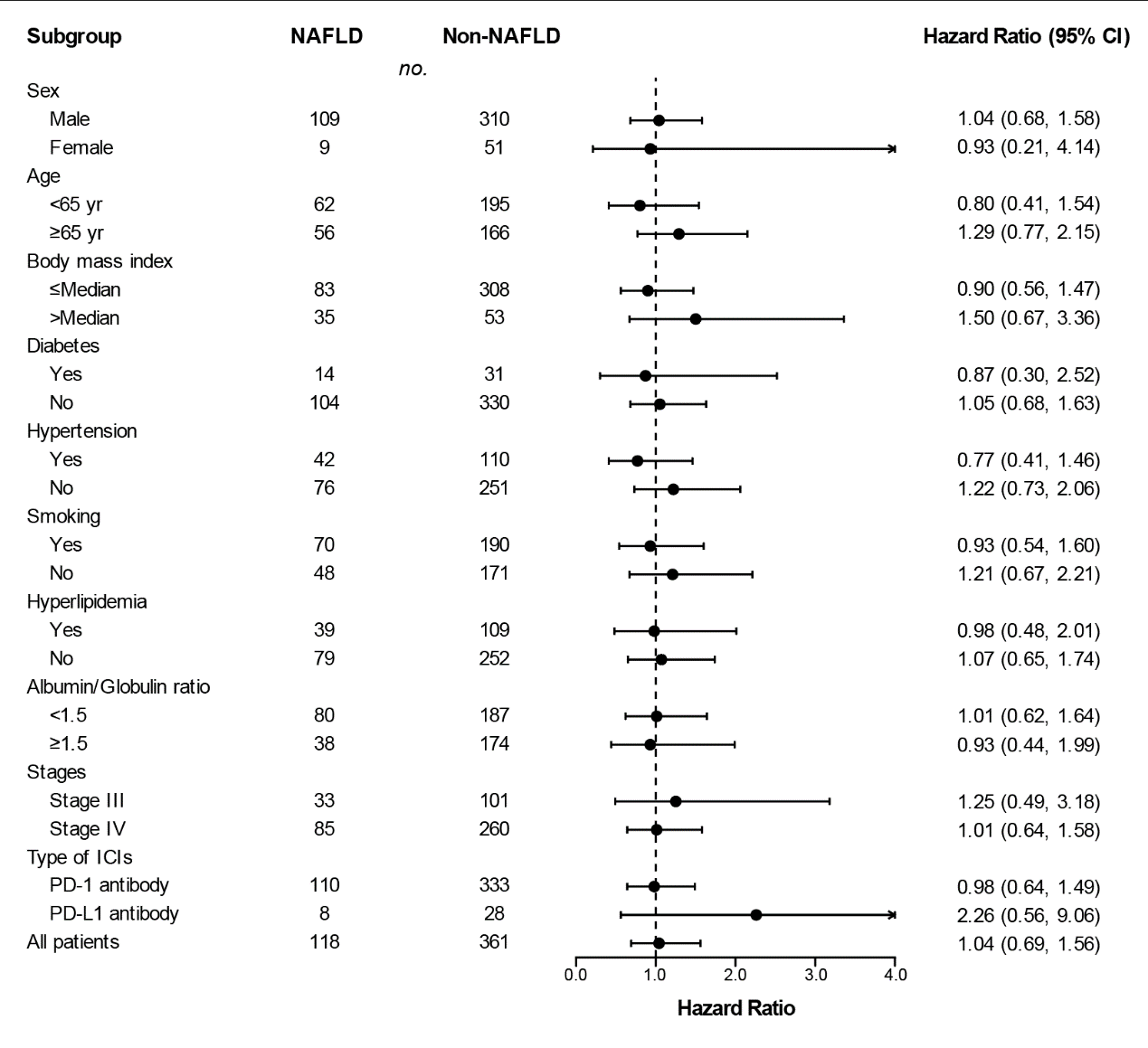


**Supplementary Figure 3** Forest plot of overall survival.
